# Adverse birth outcomes among women with ‘low-risk’ pregnancies in India: findings from the Fifth National Family Health Survey, 2019–21

**DOI:** 10.1016/j.lansea.2023.100253

**Published:** 2023-07-23

**Authors:** Ajay Tandon, Sanam Roder-DeWan, Mickey Chopra, Sheena Chhabra, Kevin Croke, Marion Cros, Rifat Hasan, Guru Rajesh Jammy, Navneet Manchanda, Amith Nagaraj, Rahul Pandey, Elina Pradhan, Andrew Sunil Rajkumar, Michael A. Peters, Margaret E. Kruk

**Affiliations:** a Global Program for Health, Nutrition and Population, World Bank, Washington, DC 20433, USA; b Harvard TH Chan School of Public Health, Harvard University, Boston, MA 02115, USA

**Keywords:** Newborn mortality, Health system quality, Risk stratification, Pregnancy risk, Health system, India

## Abstract

**Background:**

Despite substantial progress in improving maternal and newborn health, India continues to experience high rates of newborn mortality and stillbirths. One reason may be that many births happen in health facilities that lack advanced services—such as Caesarean section, blood transfusion, or newborn intensive care. Stratification based on pregnancy risk factors is used to guide ‘high-risk’ women to advanced facilities. To assess the utility of risk stratification for guiding the choice of facility, we estimated the frequency of adverse newborn outcomes among women classified as ‘low risk’ in India.

**Methods:**

We used the 2019–21 Fifth National Family Health Survey (NFHS-5)—India’s Demographic and Health Survey—which includes modules administered to women aged 15–49 years. In addition to pregnancy history and outcomes, the survey collected a range of risk factors, including biomarkers. We used national obstetric risk guidelines to classify women as ‘high risk’ versus ‘low risk’ and assessed the frequency of stillbirths, newborn deaths, and unplanned Caesarean sections for the respondent’s last pregnancy lasting 7 or more months in the past five years. We calculated the proportion of deliveries occurring at non-hospital facilities in all the Indian states.

**Findings:**

Using data from nearly 176,699 recent pregnancies, we found that 46.6% of India’s newborn deaths and 56.3% of stillbirths were among women who were ‘low risk’ according to national guidelines. Women classified as ‘low risk’ had a Caesarean section rate of 8.4% (95% CI 8.1–8.7%), marginally lower than the national average of 10.0% (95% CI 9.8–10.3%). In India as a whole, 32.0% (95% CI 31.5–32.5%) of deliveries occurred in facilities that were likely to lack advanced services. There was substantial variation across the country, with less than 5% non-hospital public facility deliveries in Punjab, Kerala, and Delhi compared to more than 40% in Odisha, Madhya Pradesh, and Rajasthan. Newborn mortality tended to be lower in states with highest hospital delivery rates.

**Interpretation:**

Individual risk stratification based on factors identified in pregnancy fails to accurately predict which women will have delivery complications and experience stillbirth and newborn death in India. Thus a determination of ‘low risk’ should not be used to guide women to health facilities lacking key life saving services, including Caesarean section, blood transfusion, and advanced newborn resuscitation and care.

**Funding:**

10.13039/100000865Bill and Melinda Gates Foundation and the World Bank. The findings, interpretations and conclusions expressed in the paper are entirely those of the authors, and do not represent the views of the Gates Foundation or of the World Bank, its Executive Directors, or the countries they represent.

**Research in context**

**Evidence before this study**

While at the population level certain maternal risk factors have long been associated with worse pregnancy outcomes, meta-analyses and individual studies at the global level note the failure of risk prediction models to identify pregnancies with adverse outcomes at the individual level. A systematic review of risk prediction models for maternal mortality concluded that neither of the top performing indices “has sufficient discrimination to be applicable to clinical decision making at the individual level” (Aoyama et al. 2018). Similarly, models based on prenatal and predelivery data (as in this study) had low prediction accuracy for perinatal and newborn mortality. An analysis of over 10 million birth records in the USA found that 29% of women classified as ‘low risk’ by stringent criteria developed an unexpected complication. Existing (small) studies in India focusing on select districts/states estimate that between 18 and 41% of all pregnancies are ‘high-risk’ pregnancies and found that these women had a higher probability of newborn complications.

**Added value of this study**

To the best of our knowledge, this is the first study in India that looks at nation-wide population-level differences in neonatal outcomes according to pregnancy risk. The study is based on a recent, large, nationally representative sample of women with recent pregnancies that allows for estimation of risk while also providing comparable data on newborn outcomes.

**Implications of all the available evidence**

In conjunction with findings from previous clinical and epidemiologic research, the current study provides strong evidence that risk stratification based on factors identified in pregnancy fails to accurately predict which women will have delivery complications and experience stillbirth and newborn death. A determination of ‘low risk’ should thus not be used to guide women to health facilities lacking key life saving services, including Caesarean section, blood transfusion, and advanced newborn resuscitation and care.

## Introduction

India—recently named the world’s most populous country—has made significant improvements in coverage of reproductive, maternal, neonatal, and child health services in recent decades. Although India still has the largest number of children born every year—some 23 million—recent survey data indicate a below-replacement total fertility rate of 2.0.^1^^,^^2^ Almost nine out of every 10 women with pregnancies in the past five years had at least one antenatal care visit and delivered at a health facility.^1^ The dramatic shift from home to facility deliveries in the last 15 years—from a 38.7% institutional delivery rate in 2005–06 to 88.6% in 2019–21—has in part been due to conditional cash transfers implemented under the *Janani Suraksha Yojana* (JSY) (Safe Motherhood Scheme). This scheme has been targeting poor and rural areas since 2005 and is now part of the National Health Mission (NHM) reform program.^3^ In 2011, JSY was augmented by *Janani Shishu Suraksha Karyakaram* (JSSK), a maternal and child protection scheme which provides free services—including transport, food, medicines, and diagnostics—to pregnant women including for normal deliveries and Caesarean-sections (C-sections) and sick newborn care for up to 30 days after birth in government health facilities. In addition to central government programs, several states have implemented additional reforms to increase hospital deliveries: e.g., Gujarat’s *Chiranjeevi (long-life) Yojana* (CY) is a health insurance scheme that reimburses private providers for providing delivery and emergency obstetric and neonatal services to poor and tribal women, and Chattisgarh’s *Dr Khoobchand Baghel Swastyha Sahayata Yojana* (DJBSSY) is a health care plan that reimburses public and empaneled private hospitals for similar services for poor women and delivery huts and birth waiting rooms for facilitating institutional deliveries in remote areas such as tribal areas. Similarly, Tamil Nadu’s Dr. Muthulakshmi Reddy Maternity Benefit Fund provided additional payments beyond JSY to women below the poverty line to deliver in facilities.

Despite improvements in coverage, maternal and newborn deaths remain above the Sustainable Development Goal (SDG) targets, with newborn mortality at 22 deaths per 1000 live births compared to the SDG target of 12, with the rate of decline slowing in recent years.^4^ National averages obscure large variation across states. Newborn mortality rates are highest in Uttar Pradesh at 36 per 1000 followed by Bihar at 35 and lowest in Kerala at 3 per 1000 live births. Maternal mortality is estimated to be 103 per 100,000 live births, far above the SDG target of 70.^5^ In 2020, nearly 24,000 women are estimated to have died from maternal causes, primarily in the poorest states.^6^ Given the current trajectory, the country as a whole, and particularly the poorest states, will not achieve the SDGs.

There are several reasons for the recent stagnation in maternal and newborn health outcomes. Health is a ‘state subject’ in India and some states have not prioritized financing for health with the resulting gaps in infrastructure and human resources. While some states, such as Kerala and Tamil Nadu, have near universal institutional delivery rates, other states continue to lag with <80% facility births, such as Bihar, Jharkhand, Meghalaya, and Nagaland. Approximately 13% of all births occurred among adolescent girls, who tend to have worse outcomes. Quality of care remains a major problem: the Lancet Global Health Commission on High Quality Health Systems estimated that 66% of all deaths amenable to medical care in India occur because of poor quality as opposed to non-utilization.^7^ Coverage rates have increased, yet *effective* coverage remains low. Thus while the proportion of facility births has grown, many deliveries occur in facilities without capacity for definitive treatment of maternal or newborn complications.^8^ Immediate, expert treatment of complications such as hemorrhage or asphyxia is needed for maternal and newborn survival during delivery; previous research has shown that this cannot be consistently accomplished at primary care facilities.^9^^,^^10^

To address some of these challenges, in 2016 India launched the *Pradhan Mantri Surakshit Matritva Abhiyan* (PMSMA) program. PMSMA is the Prime Minister’s Safe Motherhood Campaign, which aims to provide free universal comprehensive high-quality antenatal care by a physician/specialist (including by encouraging voluntary provision from private doctors) to all pregnant women at least once during their second or third trimester at designated public facilities. One of the key tenets of PMSMA is to risk stratify pregnancies and to ensure that all ‘high-risk’ pregnancies deliver in a facility that provides assured comprehensive emergency obstetric and newborn care.^11^ PMSMA is similar to efforts in many low- and middle-income to identify the subset of women who should deliver in higher-versus lower-level facilities. Although steering women to different levels of the health system based on risk is an understandable policy response to the challenge of shifting millions of deliveries from home to health facilities, it may undermine India’s ambition to reduce its maternal and newborn mortality if a significant proportion of ‘low-risk’ pregnancies go on to have adverse birth outcomes.

In this paper, we use the latest national household survey to compare the frequency of stillbirths and newborn deaths between pregnancies classified as ‘low risk’ and ‘high risk’ in India based on local PMSMA guidelines. We further analyze the proportion of ‘low risk’ pregnancies that result in an unplanned C-section, a proximal signal of the failure of risk prediction. Finally, we calculate the proportion of women who deliver at lower-level facilities versus hospitals across Indian states to assess the magnitude of a potential shift in care models. We discuss policy implications for India’s health system and potential ways forward.

## Methods

### Data sources

We use the 2019–21 Fifth National Family Health Survey (NFHS-5)—India’s Demographic and Health Survey—for which most indicators are representative at the national, state (for 28 states and eight union territories), and district levels.^1^ NFHS-5 is a household survey which collects information on demographics and socioeconomic status and includes a separate module administered to women 15–49 years of age on their reproductive history including children ever born, detailed birth and pregnancy history for the five years prior to survey, and current pregnancy status. In addition, the survey collects several biomarkers including height, weight, hemoglobin, blood glucose, and blood pressure during the time of the survey. For assessing trends in facility deliveries, we analyzed data from previous iterations of NFHS data (from 1992–93, 1998–99, 2005–06, and 2015–16) which all follow a similar structure to that of NFHS-5.

### Data analysis

We examined three categories of unexpected complications and adverse birth outcomes during their latest pregnancy as reported by women 15–49 years of age in the five years preceding the survey: unplanned C-section, stillbirth, and neonatal death (Table 1). Unplanned C-sections were self-reported deliveries by C-section where the decision to perform the procedure was made *after* the onset of labor. The percent of unplanned C-section is calculated as number of unplanned C-sections divided by total number of births multiplied by 100. Stillbirths were identified using self-reported pregnancy histories as fetal deaths that occurred in pregnancies that lasted seven or more months. Neonatal deaths were deaths that occurred among newborns within 28 days of birth. The rate of stillbirths and neonatal deaths is calculated as the number of the observed outcome divided by total number of pregnancies multiplied by 1000 live births.

**Table 1:** Definitions of unexpected complications and adverse birth outcome indicators.

Indicator	Definition
Unplanned C-section	Deliveries by C-section with decision made to have the C-section after onset of labor
Stillbirths	Pregnancy losses that occurred after seven or more months of gestation
Neonatal deaths	Death of newborns within 28 days of birth

If both a live birth and a stillbirth occur together, only the live birth is recorded which may lead to some undercounting of stillbirths. For the most recent pregnancy which ended in a stillbirth, the NFHS-5 data collection structure precluded collection of the following variables: (i) multiple pregnancy, (ii) breech presentation, (iii) first antenatal visit after six months of pregnancy, and (iv) institutional delivery.

All estimates were made after indicating the survey structure using the “svyset” command in Stata version 17.0. This command specifies the individual, household, and cluster sampling and post-stratification weights provided by the NFHS as well as survey strata. Point estimates and associated confidence intervals thus incorporate the complex survey design of the NFHS and post-stratification weights.

We derived conditions used to risk stratify a pregnancy from the PMSMA, which consolidates India’s national treatment guidelines. Under PMSMA, conditions for classification as a ‘high-risk’ pregnancy include severe anemia (Hb < 7 gm/dl); pregnancy-induced hypertension; pre-eclampsia; pre-eclamptic toxemia; syphilis/HIV; gestational diabetes; hypothyroidism; mother’s age <20 or >35; multiple pregnancy; malpresentation; previous lower-segment C-section; low-lying placenta; placenta previa; history of (other) obstetric complication including stillbirth, abortion, congenital malformation, obstructed labor, or premature birth; Rhesus (Rh) factor negative; intrauterine growth restriction; or patients with history of any current systemic illness(es)/past history of illness.^11^

Based on the above list, we identified a subset of variables for which information was available from the NFHS-5 survey, either at the time the survey was administered (e.g., whether the woman was hypertensive, diabetic, or had other co-morbidities such as chronic respiratory disease including asthma, thyroid disorder, heart disease, cancer, or chronic kidney disorder) or at the time of last birth (e.g., mother’s age at birth or birth order). Women with one or more of the risks identified under PMSMA available in NFHS-5—severe anemia, hypertension, diabetes, other co-morbidities, maternal age, multiple pregnancies, breech presentation, previous C-section, previous adverse birth outcomes—are reported as ‘high risk’ (Table 2).

**Table 2:** Pregnancy risk factors based on India’s PMSMA guidelines.

PMSMA pregnancy risk factor	Derivation from NFHS-5
Hypertension	Average of three blood pressure readings with systolic blood pressure >130 mm Hg and/or diastolic blood pressure >85 mm Hg and/or currently on blood pressure medication at time of survey
Diabetes	Random blood glucose level ≥200 mg/dl and/or currently on diabetes medication at time of survey
Other comorbidity	Self-report response to question
Maternal age <20	Based on mother’s age during last pregnancy
Multiple pregnancy	Self-report whether last pregnancy was multiple
Breech presentation	Self-report whether breech presentation during last delivery
Previous C-section delivery	Based on pregnancy history
Previous stillbirth or neonatal death	Derived from pregnancy history
Severe anemia (Hb < 7 gm/dl)	Based on blood samples taken and analyzed at time of survey
Maternal age >35	Based on mother’s age during last pregnancy

The presence of one or more risk factor classifies a pregnancy as ‘high risk’.

We also evaluated where women deliver across the country using current and previous NFHS surveys. NFHS-5 captures place of delivery reported by women respondents and we use these data to infer the proportion who deliver in facilities equipped to provide emergency obstetric and newborn care. In terms of public facilities, although some Community Health Centers (CHCs) are meant to provide emergency obstetric care services (including surgeries), in reality many do not.^12^ First referral units (FRUs) in such cases are often the sub-district or district hospital.^8^ Because the NFHS groups several types of facilities together, following Gage and colleagues, we categorize NFHS facility categories as “public non-hospitals” if the share of C-sections among all deliveries was <10%.^13^ This was the case for CHCs, rural hospitals, block primary health centers (PHCs), PHCs, additional PHCs, and sub-centers (Appendix Table S1).^13^ All other public facility categories, including government/municipal hospitals, urban health centers (UHCs)/urban health posts (UHPs)/urban family welfare centers (UFWCs), had C-section rates among deliveries ≥10% and were categorized as public hospitals/higher-level facilities.^13^ Private facilities were separately categorized, as were women who delivered at home. We further assessed several demographic characteristics of women who deliver in hospitals versus non-hospitals (mother’s education, economic status, rural-urban residence, and whether they belonged to a scheduled caste or scheduled tribe).

We conducted an additional sensitivity test to see if more rigorous risk-stratification would change the results. Danilack and colleagues adopt a narrow definition of ‘low-risk’ pregnancies; women were classified as ‘high risk’ if they met one or more of 19 criteria. These criteria were derived from a variety of sources including guidance documents from the American College of Obstetricians and Gynecologists, the clinical literature, and U.S. National Vital Statistics population data on maternal characteristics associated with fetal and perinatal mortality. We applied the Danilack criteria to illustrate the impact of more rigorous prenatal risk stratification on the outcomes of interest. For categorization as ‘high risk’ by these more stringent criteria, we identified additional NFHS-5 variables corresponding to selected risk factors included in the paper by Danilack et al. (2015). These were: previous pregnancy interval <15 months, body mass index (BMI) ≥30, and if the first antenatal visit happened after six months of pregnancy. Information on other risks identified by PMSMA or in Danilack et al. (2015)—e.g., syphilis/HIV, hypothyroidism, Rh negative, cervical cerclage, premature rupture of membranes, tocolysis, congenital anomalies, pregnancy-induced hypertension, pre-eclampsia, pre-eclamptic toxemia, gestational diabetes, low-lying placenta, placenta previa, history of obstructed labor and premature birth, and hepatitis B or C—were not available in NFHS-5. These additional estimates also accounted for the complex survey structure and all weights.

## Results

**Fig. 1:**
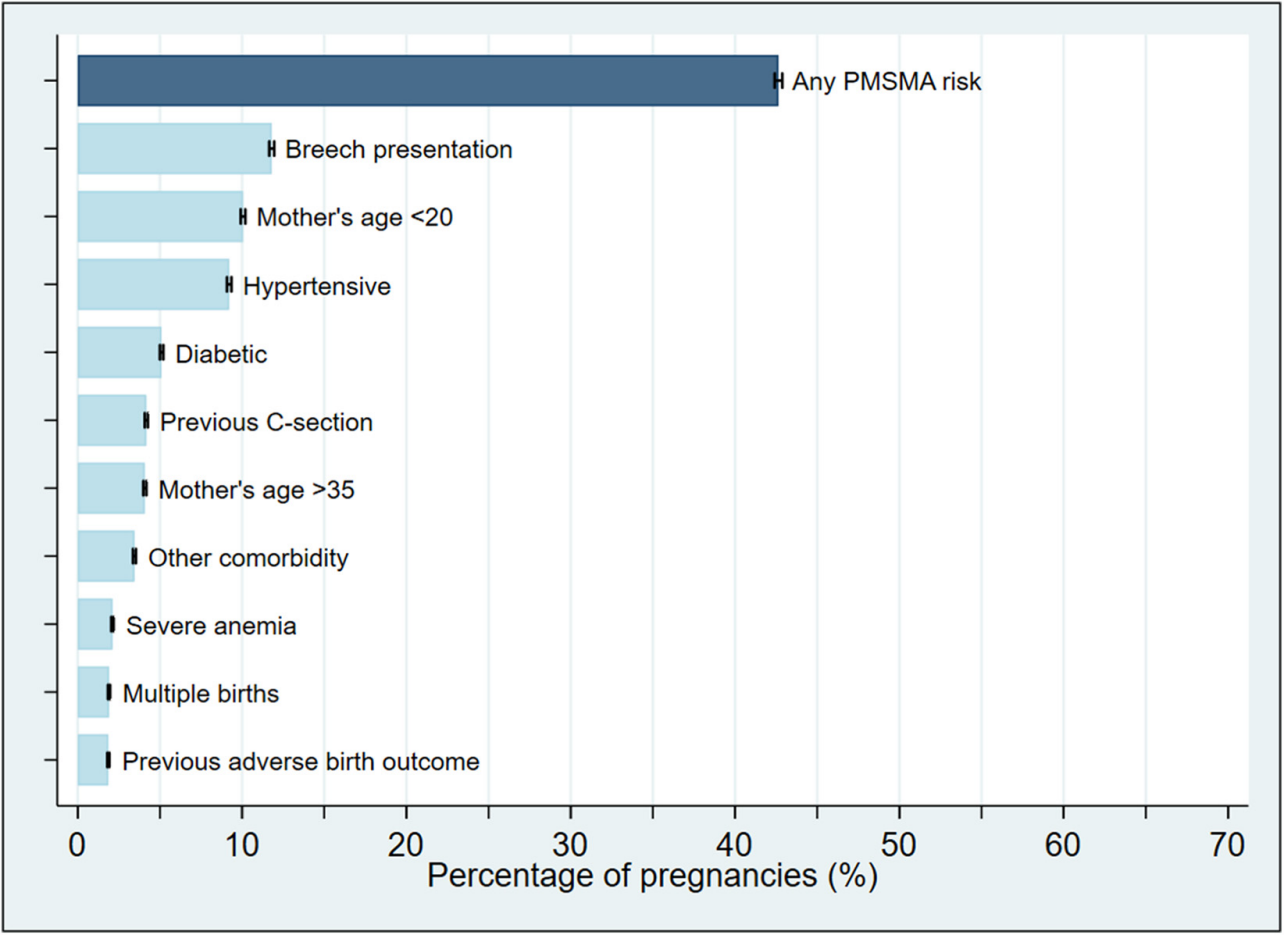
Prevalence of risk factors in the last pregnancy that lasted 7+ months among women aged 15–49.

NFHS-5 interviewed a total of 636,699 households, including 724,115 eligible women aged 15–49 years of age with a response rate of 98%. Among those eligible, there were a total of 176,699 pregnancies that lasted 7+ months in the five years preceding the survey. The median age of the latest (youngest) living child among these women was just under two years (23 months). Baseline characteristics of mothers in low- and high-risk groups were roughly similar (Table 3). 42.6% (95% CI 42.4–42.9%) of pregnancies had at least one of the risk factors identified under the PMSMA program for which data were available in NFHS-5 (Fig. 1). The most common risk factors were experiencing a breech presentation (11.8% of pregnancies, 95% CI 11.5–12.1%) and being less than 20 years of age at the time of birth (10.1% of pregnancies, 95% CI 9.8–10.3%).

**Table 3:** Baseline characteristics of study respondents.

Demographics	Total	Low-risk	High-risk
	N = 176,699	N = 101,348	N = 75,351
Age at time of survey			
15–19	3% (5562)	0% (0)	7% (5562)
20–24	29% (51,716)	27% (27,438)	32% (24,278)
25–29	39% (68,410)	45% (45,938)	30% (22,472)
30–34	20% (34,695)	22% (22,273)	16% (12,422)
35–39	7% (12,753)	6% (5699)	9% (7054)
40–44	2% (2897)	0% (0)	4% (2897)
45–49	<1% (666)	0% (0)	1% (666)
Missing	0% (0)	0% (0)	0% (0)
Place of residence			
Urban	28% (49,877)	28% (28,705)	28% (21,171)
Rural	72% (126,822)	72% (72,642)	72% (54,180)
Missing	0% (0)	0% (0)	0% (0)
Highest educational level			
No education	20% (34,496)	20% (20,348)	19% (14,111)
Primary	12% (20,693)	12% (12,251)	11% (8441)
Secondary	51% (90,937)	50% (50,265)	54% (40,672)
Higher	17% (30,573)	18% (18,447)	16% (12,126)
Missing	0% (0)	0% (0)	0% (0)
Wealth index			
Poorest	23% (40,248)	23% (23,174)	23% (17,074)
Poorer	21% (37,171)	21% (21,268)	21% (15,903)
Middle	20% (34,550)	19% (19,398)	20% (15,152)
Richer	19% (33,983)	19% (19,410)	19% (14,572)
Richest	17% (30,747)	18% (18,097)	17% (12,650)
Missing	0% (0)	0% (0)	0% (0)
Caste			
Schedule caste	22% (39,601)	22% (22,789)	22% (16,812)
Schedule tribe	10% (17,317)	10% (10,199)	9% (7119)
Other backwards caste	43% (75,405)	43% (43,917)	42% (31,488)
None of them	19% (32,768)	18% (18,689)	19% (14,079)
Don't know	1% (1551)	1% (774)	1% (777)
Missing	6% (10,057)	5% (4980)	7% (5077)
Individual risk factors			
Has severe anemia	2% (3731)	0% (0)	5% (3731)
Missing	5% (8291)	5% (5408)	4% (2882)
Has high blood pressure	9% (16,278)	0% (0)	22% (16,278)
Missing	4% (6449)	4% (4461)	3% (1988)
Has high glucose	5% (9014)	0% (0)	12% (9014)
Missing	5% (8122)	5% (5310)	4% (2812)
BMI			
18.5 > BMI > 30	74% (130,295)	74% (74,806)	74% (55,489)
BMI ≤ 18.5	18% (31,913)	18% (18,534)	18% (13,379)
BMI ≥ 30	8% (14,491)	8% (8008)	9% (6483)
Missing	0% (0)	0% (0)	0% (0)
Has other comorbidity	3% (6095)	0% (0)	8% (6095)
Missing	0% (0)	0% (0)	0% (0)
Pregnancy history			
Pregnancy interval			
First pregnancy	32% (56,442)	30% (30,224)	35% (26,217)
Last pregnancy <15 months ago	17% (29,630)	16% (16,379)	18% (13,252)
Last pregnancy ≥15 months ago	51% (90,626)	54% (54,744)	48% (35,882)
Missing	0% (0)	0% (0)	0% (0)
Breech presentation	12% (20,857)	0% (0)	28% (20,857)
Missing	4% (7752)	4% (3556)	6% (4196)
Previous C-section delivery	4% (7364)	0% (0)	10% (7364)
Missing	<1% (492)	<1% (293)	<1% (199)
Multiple pregnancy	2% (3357)	0% (0)	4% (3357
Missing	1% (1501)	1% (845)	1% (657)
Previous stillbirth or neonatal death	2% (3293)	0% (0)	4% (3293)
Missing	0% (0)	0% (0)	0% (0)

Totals and proportions are weighted for individual probability of selection and account for the complex survey design.

The neonatal mortality rate estimated from the last birth in previous five years was 16.6 per 1000 live births (95% CI 15.9–17.4%). 10.0% (95% CI 9.8–10.3%) of all pregnancies resulted in an unplanned C-section (Table 4); there were 25.1 stillbirths or neonatal deaths per 1000 live births (95% CI 24.2–26.1); 8.4% (95% CI 8.1–8.7%) of pregnancies that would be classified as ‘low risk’ under PMSMA went on to have an unplanned C-section. Furthermore, there were 21.9 (95% CI 20.7–23.1) adverse outcomes per 1000 live births among PMSMA ‘low-risk’ pregnancies. Despite the share of adverse birth outcomes being higher among ‘high-risk’ women (as might be expected), 49.8% of all adverse birth outcomes that occurred were among women that were classified as ‘low risk’ under PMSMA (Table 4).

**Table 4:** Frequencies of unplanned C-sections, stillbirths, and neonatal deaths in the last pregnancy that lasted 7+ months among women aged 15–49.

Outcome	Unplanned C-sections^a^ *N* (%)	Stillbirths^b^ *N* (# per 1000 livebirths)	Neonatal deaths^c^ *N* (# per 1000 livebirths)	Composite mortality^d^ *N* (# per 1000 livebirths)
Among all pregnancies [*N* = 176,699]	17,717 (10.0%) (95% CI 9.8–10.3%)	1504 (8.5) (95% CI 7.9–9.1)	2940 (16.6) (95% CI 15.9–17.4)	4444 (25.1) (95% CI 24.2–26.1)
*Any PMSMA risk*				
Among low-risk pregnancies *N* = 101,347 (57.4%)	8512 (8.4%) (95% CI 8.1–8.7%)	846 (8.3) (95% CI 7.6–9.1)	1369 (13.5) (95% CI 12.6–14.5)	2215 (21.9) (95% CI 20.7–23.1)
Among high-risk pregnancies *N* = 75,351 (42.6%)	9205 (12.2) (95% CI 11.9–12.6%)	659 (8.7) (95% CI 7.9–9.7)	1571 (20.8) (95% CI 19.5–22.2)	2229 (29.6) (95% CI 28.0–31.3)
Percentage of outcome from low-risk pregnancies	48.0%	56.3%	46.6%	49.8%

a Unplanned C-sections are those that were not planned before the mother was in labor.

b Stillbirths are pregnancies where the fetus dies after the mother’s 28th week of pregnancy. Due to data collection structure of NFHS, multiple pregnancy and breech presentation variables are not available for stillbirth calculation.

c Neonatal deaths are deaths among live births during the first 28 completed days of life.

d Composite is the summation of stillbirths and neonatal deaths. Data presented are the weighted estimates from the sample and rates are estimated per 1000 live births along with 95% confidence intervals that account for the complex survey design.

When additional risk factors from the global literature were added in the sensitivity analysis, 57.7% (95% CI 57.5–57.9%) had at least one risk factor: e.g., 16.8% (95% CI 16.6–16.9%) were pregnancies which had a short pregnancy interval (<15 months), 8.2% (95% CI 8.1–8.3%) were to women with BMI ≥30, and 4.6% (95% CI 4.5–4.7%) were pregnancies where the first ANC visit occurred after six months into the pregnancy (Appendix Table S2). Results were similar to the PMSMA analysis: even with more rigorous risk classification criteria, the stillbirth rate and neonatal mortality rate were similar between low- and high-risk groups.

**Fig. 2:**
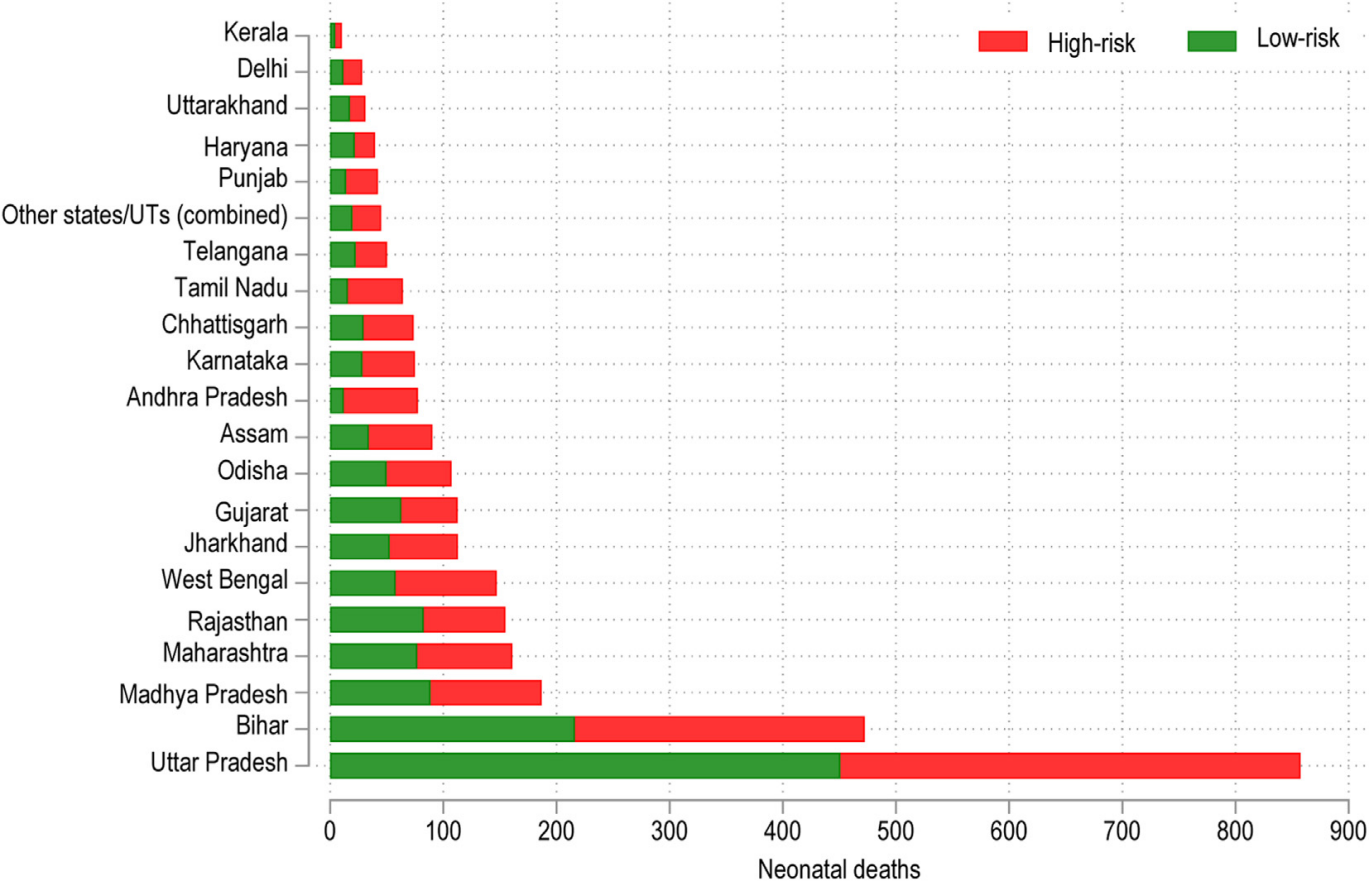
Number of neonatal deaths for selected states and union territories by PMSMA risk category in the last pregnancy that lasted 7+ months among women aged 15–49, 2019–21.

Source: Authors’ calculation from NFHS-5.

There are large variations in newborn mortality and stillbirths by state (Fig. 2). States such as Kerala and Tamil Nadu have much lower neonatal mortality rates—for both ‘low-risk’ and ‘high-risk’ pregnancies—relative to states such as Bihar and Uttar Pradesh: the neonatal mortality rate for the latest birth prior to the survey was 2.6 per 1000 live births in Kerala and 7.3 in Tamil Nadu; compared to 23.2 in Bihar and 25.9 in Uttar Pradesh. In most states nearly half of all neonatal deaths occurred among women who would be classified as ‘low risk’ under the PMSMA scheme (see Appendix Table S3 for state-level data).

**Fig. 3:**
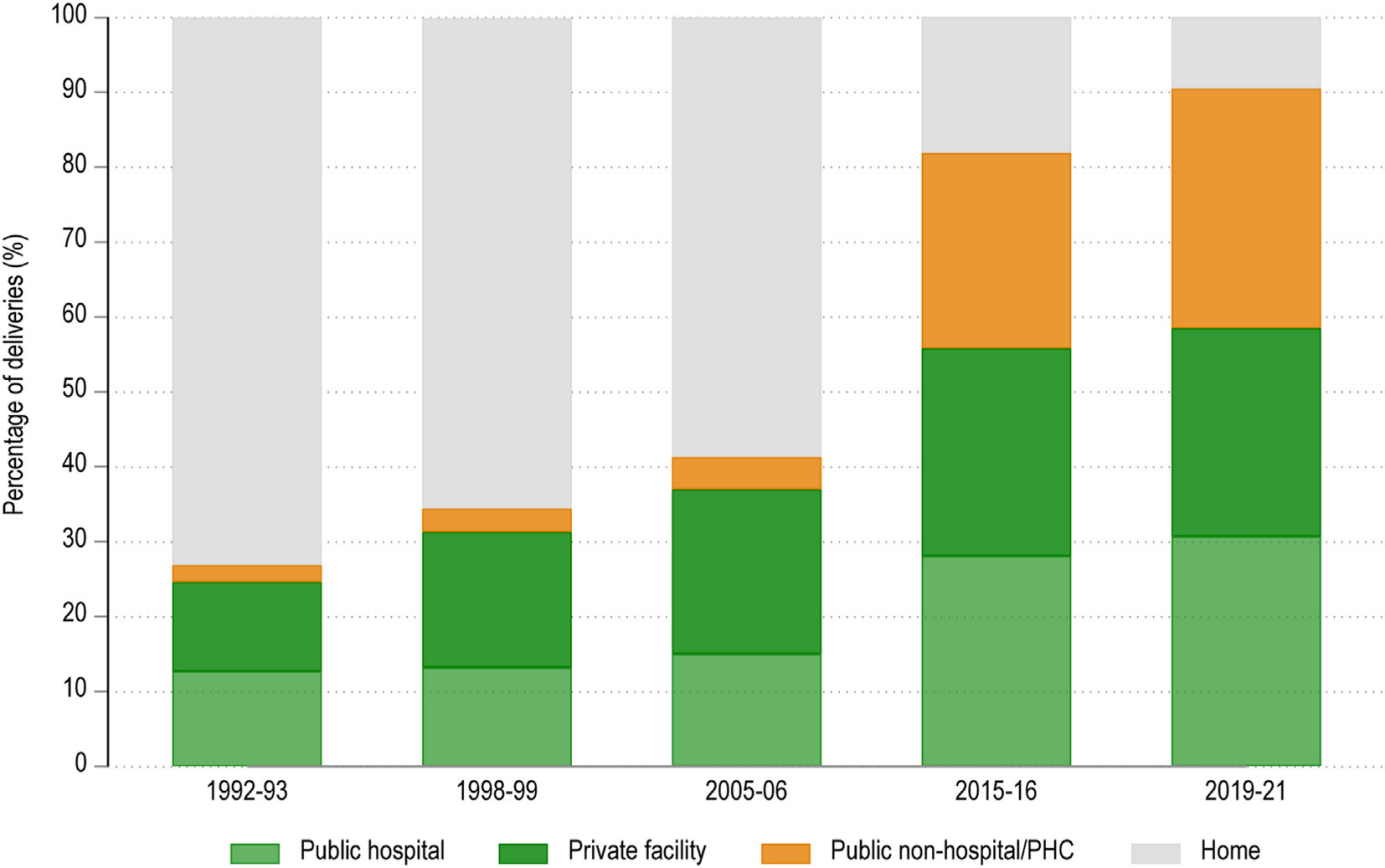
Place of delivery among women aged 15–49 time trends from successive waves of NFHS.

Source: Authors’ calculation from NFHS (various years).

Across all pregnancies, we found that 32.0% (95% CI 31.5–32.5%) of deliveries occurred at lower-level facilities, 30.7% (95% CI 30.2–31.2%) at public hospitals, 27.8% (95% CI 27.3–28.2%) at private facilities, and 9.6% (95% CI 9.3–9.8%) at home. 32.1% (95% CI 31.9–32.4%) of ‘low-risk’ pregnancies occurred at public non-hospitals/PHCs (lower-level facilities), and of all pregnancies that occurred at lower-facilities 60.2% (95% CI 59.8–60.6%) were to women who would be classified as ‘low risk’ under PMSMA guidelines. Among all the births that occurred at ‘public non-hospital/PHC’ facilities in 2019–21, most were to women of relatively lower socioeconomic status and background: 87.6% (95% CI 87.1–88.1%) were rural, 62.6% (95% CI 62.0–63.2%) were from the bottom two economic quintiles, 40.3% (95% CI 39.5–41.1%) were women belonging to a scheduled caste or scheduled tribe, and 49.3% (95% CI 48.7–48.0%) had seven or fewer years of schooling. Fig. 3 shows time trends in the use of non-hospitals for delivery across the country, based on successive NFHS survey waves. We found that the share of births occurring at such lower-level facilities rose from 2.3% (95% CI 2.0–2.6%) in 1992–93 to 32.0% (95% CI 31.5–32.5%) in 2019–21 (Fig. 3). This level of the health system saw the largest increase in use over this time.

**Fig. 4:**
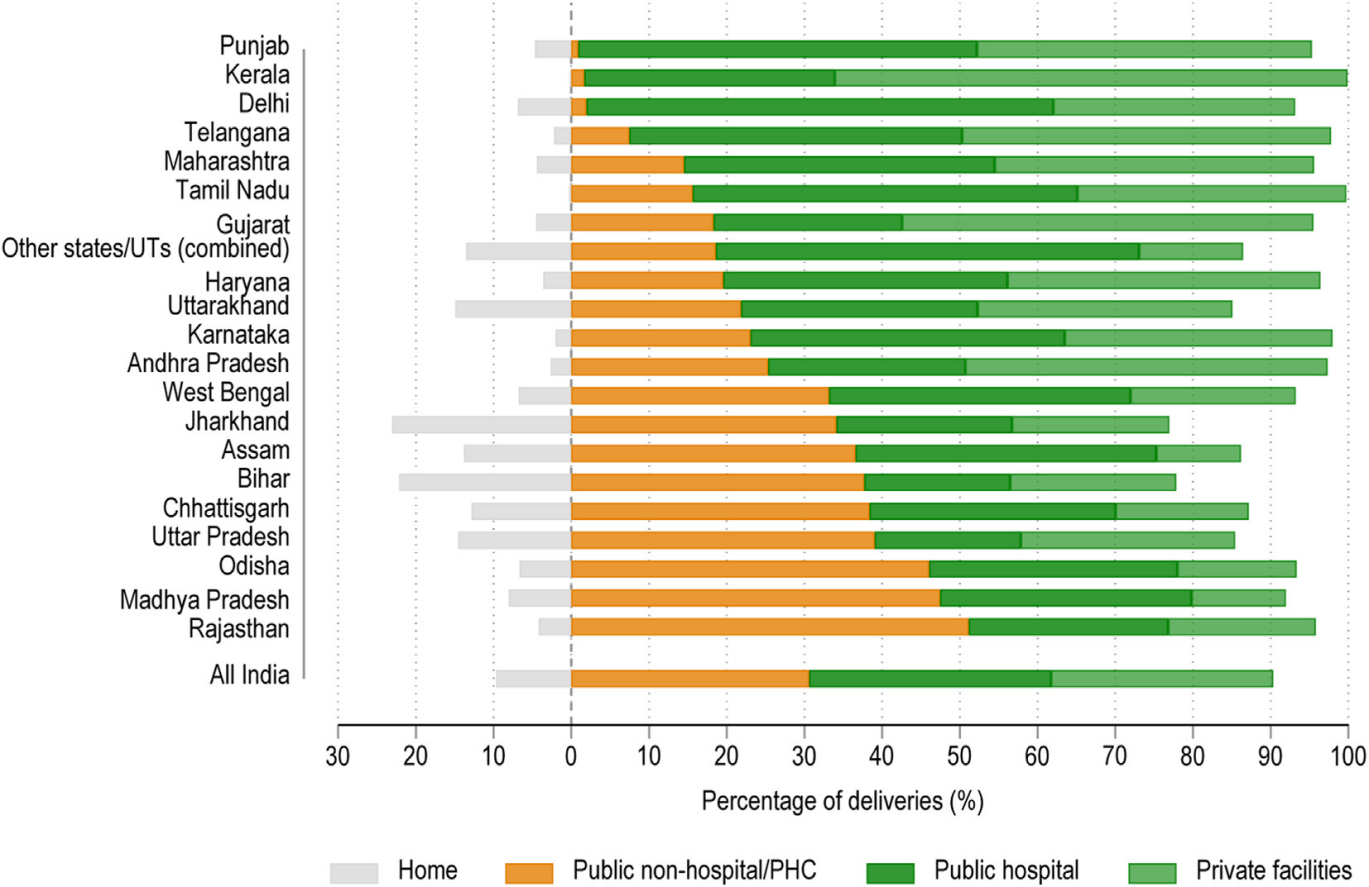
Place of delivery across states in India among women aged 15–49, 2019–21.

Source: Authors’ calculation from NFHS-5.

Fig. 4 shows the distribution of place of delivery in the Indian states. Across India, 9.6% (95% CI 9.3–9.8%) of births occurred at home. In some states such as Bihar and Jharkhand, home deliveries continue to exceed one-fifth of all deliveries. There are large variations in the composition of facility-based deliveries. Kerala and Gujarat have the highest share of births occurring at private facilities; Assam and Madhya Pradesh have the lowest. The share of public lower-level facilities among institutional deliveries is highest in states such as Rajasthan, Madhya Pradesh, Uttar Pradesh, Bihar, Odisha, Assam, and West Bengal. These are also states with some of the lowest private sector delivery shares.

## Discussion

Using recent nationally representative survey data from India, we found that almost half of India’s newborn deaths and more than half of stillbirths were among women who were ‘low risk’ according to national guidelines. We found that 42.6% (95% CI 42.4–42.9%) of pregnancies were classified as ‘high risk’ using national PMSMA criteria. While, as expected, the likelihood of neonatal mortality and stillbirth was higher among ‘high-risk’ women, babies born to ‘low risk’ women were a major contributor to India’s newborn deaths and stillbirths. This suggests that stratifying pregnancies based on risk factors is not effective in predicting perinatal or newborn outcomes. Furthermore, we found that among ‘low-risk’ women, 8.4% (95% CI 8.1–8.7%) had an unplanned Caesarean section, only slightly less than the national average of 10.0% (95% CI 9.8–10.3%). This service is not provided in most lower level facilities. Taken together, these data indicate that the lack of pregnancy risk factors should not be used to direct women to deliver in lower level facilities.

Whereas at the population level pregnancy risk factors are associated with worse birth outcomes, recent studies have found that it is not possible to accurately predict the risk of unexpected complications and adverse birth outcomes at the individual level from information available during the pregnancy. A systematic review of risk prediction models for maternal mortality concluded that even the top performing indices did not have “sufficient discrimination to be applicable to clinical decision making at the individual level”.^14^ Similarly, models based on prenatal and predelivery data (as in this study) had low prediction accuracy for perinatal and newborn mortality.^15^ An analysis of over 10 million birth records in the USA found that 29% of women classified as ‘low-risk’ under stringent criteria developed an unexpected complication. Of these ‘low risk’ women, 15% had an unplanned C-section, a finding similar to ours. Thus, even with expanded criteria, in a wealthier context with wide availability of diagnostics and expert care in pregnancy, prenatal risk stratification did not reliably predict complications.^16^

For this reason, and because complications of most mothers and children often arise suddenly and require rapid treatment by expert teams, the minimum level delivery facility in high-income countries is typically a hospital with surgical care, anesthesia, blood transfusion, and newborn intensive care.^17^ While delivery in hospitals is standard practice in low mortality countries, this has not been the case in many high mortality countries, including India. In many Indian states the efforts to expand access to maternal care and reduce home births have taken the path of encouraging delivery in PHCs and CHCs, which generally lack advanced services and which we have termed “non-hospitals” in this paper.^12^^,^^18^^,^^19^ While some of these facilities are intended to provide Caesarean section and other advanced services, infrastructure and health worker shortages preclude this in practice. A recent national report noted that 80% of specialist posts, including 74% of obstetrician posts, in CHCs, were vacant.^20^

Recent evidence suggests that use of non-hospital facilities for delivery, together with severe human resource shortage for doctors and specialists, may be important factors in the stagnation of maternal and newborn mortality reduction in India. An ecologic study found the expected negative association between newborn mortality and hospital delivery but not primary care delivery in South Asia.^13^ Furthermore, experimental evidence suggests that quality improvement in primary care facilities is not sufficient to reduce mortality in India. The BetterBirth Study, a large randomized controlled trial, found that a quality improvement bundle did not reduce perinatal death, in PHCs, CHCs and sub-district hospitals (SDHs) in 24 districts in Uttar Pradesh.^1^^,^^9^ Of note was the study’s low C-section rate in intervention (1.8%) and control (1.7%) groups, rates far below the optimal C-section rate, which is around 19%. It is possible then that the trial failed to improve outcomes because it targeted a level of the health system not currently capable of immediate provision of advanced life-saving care.

We found that nearly one-third of deliveries in India occurred in facilities likely lacking advanced services (here termed non-hospitals) and that these lower level deliveries were a major driver of the rise in facility delivery over the past two decades. Women delivering in non-hospitals were poorer, less educated, and more likely to belong to a scheduled caste or scheduled tribe; this may contribute to India’s socioeconomic disparities in newborn outcomes. At the state level different obstetric care approaches have emerged, with varied results. There was substantial variation across the country, with less than 5% non-hospital facility delivery in Punjab, Kerala, and Delhi to more than 40% in Odisha, Madhya Pradesh, and Rajasthan. States with the lowest neonatal mortality rates—e.g., Kerala and Tamil Nadu—have low home delivery rates, with vast majority of deliveries at hospitals vis-à-vis lower-level facilities. Most births occur at private facilities—generally those that provide comprehensive emergency maternal, obstetric, and newborn care—in states such as Kerala, Karnataka, Andhra Pradesh, Haryana, and Gujarat. By contrast, Rajasthan, Madhya Pradesh, Uttar Pradesh, Bihar, Odisha, Assam, and West Bengal have high rates of non-hospital deliveries along with some of the highest levels of neonatal mortality.

Our findings, in conjunction with the above studies and the experience of high and most middle-income countries, suggest that shifting births to hospitals or other advanced facilities that can provide definitive care for complications should be explored to reduce newborn deaths and stillbirths and well as maternal deaths in India. To this end, the Lancet Global Health Commission on High Quality Health Systems proposed Service Delivery Redesign (SDR) to guide this shift. SDR for maternal and newborn health proposes reorganizing obstetric care so that all women (not just putatively ‘high-risk’ women) can deliver in high-quality facilities with the staff, tools, and expertise to effectively treat maternal and newborn complications while providing support for physiologic birth and respectful care.^7^^,^^21^ In addition to strengthening hospitals, investment is urgently needed in primary care to offer quality prenatal and postnatal services and in some areas, roads and transportation to improve access to the health system. Communities and providers need to have an integral role in planning and participating in the design of new care models fitted to each context.

While the core principle of rapid (within 30 min), definitive care for complications for all women should be the national minimum standard, specific care models will vary depending on geography, culture, and health system capacity around the country.^21^ Options include expanding hospital maternity services and on-site or near-site birthing centers that can rapidly transfer women to advanced care. These can be staffed by midwives or other trained professionals with support from obstetricians. For geographically isolated and sparsely populated areas of India, models of care may need to include maternity waiting homes near well-equipped hospitals or upgrades to health centers. Where private hospitals are available, state governments could follow Gujarat and Chhattisgarh in publicly financing normal and complicated deliveries for the poor and vulnerable.^2^ More generally, given its large size, the private sector should be fully engaged in any reform with regulation to guard against over-medicalization. This is a concern in India with very high Caesarean section rates in some private facilities, for example in Tamil Nadu.^22^ Regardless of the model, the needs of poor and vulnerable groups must be prioritized from the start to avoid inducing or widening existing disparities.

This study had several limitations. First, the NFHS did not have a sufficient sample size to assess maternal mortality, a vital policy area for India. Second, not all risk variables in PMSMA were available in the NFHS data. We had information on current status of women’s hypertension/diabetes/BMI, not their status during pregnancy (although limiting the analysis to the latest pregnancy could reduce some challenges with this assumption). Similarly, NFHS records the current anemia levels of women, but not their anemia status during pregnancy. About 70% of all women, regardless of current anemia level, reported receiving some supplementary nutrition from *aanganwadi* centers during their last pregnancy, but this is relatively consistent across all risk strata. The NFHS data structure also limited understanding of certain outcome variables. For example, we are unable to understand whether some C-sections planned before the onset of labor were for emergency purposes, which may underestimate the number of emergency C-sections. Furthermore, we could not fully ascertain whether specific facilities where women delivered provided advanced care as a) these were grouped across several facility types and b) as noted above, facilities sometimes do not provide advanced services in practice. We thus relied on Caesarean-section rates across the survey categories for our estimates of non-hospital delivery, following the practice of previous studies.^13^^,^^23^

The results presented here aim to contribute to the policy dialogue on India’s ongoing *Ayushman Bharat* (Long Live India) reforms that are focused on provision of comprehensive primary health care at frontline public health facilities while financing hospitalization for the poor at both public and private hospitals. For example, Pradhan Mantri Jan Arogya Yojana (People’s Health Scheme) could expand its benefits package to finance coverage of all deliveries at empaneled private facilities that provide comprehensive emergency maternal, obstetric, and newborn care and JSY could be tailored to incentivize deliveries to occur not just at any government institution but specifically at higher-level public and private facilities where such services are available. India may already be moving toward SDR—the Indian government launched the *Surakshit Matritva Ashwasan* (SUMAN) (Safe Motherhood Service Guarantee) program in 2019 that integrates all maternal and neonatal health programs, aiming to provide universal access to free, high-quality, and comprehensive maternal and newborn services. The program stipulates that all SUMAN medical colleges, district hospitals, designated CHCs, and SDHs should provide advanced obstetric services; this is in line with our findings that indicate the need to raise the standard for delivery facilities. Where higher-level facilities are not available, the government must continue to prioritize investments for upgrading existing public facilities to provide such services including investments to improve human resources for health in these facilities, or incentivize private providers to provide these advanced obstetric services.

This study, taken together with other research, points to the need for rethinking the model for obstetric care in India by making high-quality obstetric care the norm for all women, ideally starting with areas with high poverty, social exclusion, and high rates of maternal and newborn deaths. A reform of this magnitude requires high-level political leadership and effective health system management. However, over the past 20 years, India has amply demonstrated both its ambition and its capacity for health system reform.

## Contributors

MEK and AT conceptualized the study and wrote the first draft. AT conducted the analysis with support from MEK, SRO, and MAP. AT, MAP, and MEK verified underlying data. All co-authors provided input to first draft, reviewed successive drafts and made revisions. All authors approved the manuscript for submission.

## Data sharing statement

All data are in the public domain, available from the DHS Program upon request.

## Declaration of interests

Drs. Mickey Chopra, Kevin Croke, Sanam Roder-DeWan, Andrew Sunil Rajkumar, and Margaret E. Kruk hold grants or contracts from the Bill and Melinda Gates Foundation. Several authors (SRD, MC, MAP, ANB, KC, and MEK) received travel funding for a meeting where this paper was initially conceived. Authors declare no other conflicts of interest.
