# Supplementary material for: Adverse birth outcomes among women with ‘low-risk’ pregnancies in India: findings from the Fifth National Family Health Survey, 2019–21
Source: Lancet Reg Health Southeast Asia. 2023 Jul 23;15:100253. doi: 10.1016/j.lansea.2023.100253 (PMC10382663; doi:10.1016/j.lansea.2023.100253)
Supplement: Appendix Tables S1–S3 [file mmc1.docx]

**Appendix**

**Table S1: C-section rates by facility level in NFHS survey**

| **Facility** | **NFHS-5 categories** | **Percent C-section** | ***N* facility births** |
| --- | --- | --- | --- |
| Public non-hospital/PHC | CHC/rural hospital/block PHC; PHC/ additional PHC; sub-center | 7.8%  (95% CI 7.6-8.1%) | 53,633 |
| Public hospitals | Government/Municipal hospital; Government dispensary; Other public sector hospital | 24.4%  (95% CI 24.1-24.8%) | 54,525 |
| Private facilities | Private: hospital/maternity home; Other private sector hospital; NGO or trust hospital; other | 49.7%  (95% CI 49.2-50.1%) | 50,096 |
| *All facility births* |  | *26.8%*  *(95% CI 26.6-27.0%)* | *158,253* |

Note: Data are the percentages of C-section rates and 95% confidence intervals for each of the categories of institutions where deliveries took place from NFHS-5. Abbreviations: CHC = community health center; NGO = non-government organization; PHC = primary health center;

**Table S2: Frequencies of unplanned C-sections, stillbirths, and neonatal deaths in the last pregnancy that lasted 7+ months among women aged 15-49 among Danilack high-risk pregnancies**

| **Outcome** | **Unplanned C-sections^a^**  ***N* (%)** | **Stillbirths^b^**  ***N* (# per 1,000 live births)** | **Neonatal deaths^c^**  ***N* (# per 1,000 live births)** | **Composite mortality^d^**  ***N* (# per 1,000 live births)** |
| --- | --- | --- | --- | --- |
| Among all pregnancies  [*N*=176,699] | 17,717 (10.0%) (95% CI 9.9-10.2%) | 1,504 (0.9%)  (95% CI 0.8-0.9%) | 2,940 (1.7%)  (95% CI 1.6-1.7%) | 4,444 (2.5%)  (95% CI 2.4-2.6%) |
| *Any Danilack Risk* | | | | |
| Among low-risk pregnancies  *N*=74,736 (42.3%) | 6,465 (8.7%)  (95% CI 8.4-9.0%) | 665 (8.9)  (95% CI 8.0-9.9) | 934 (12.5)  (95% CI 11.5-13.5) | 1,599 (21.4)  (95% CI 20.1-22.8) |
| Among high-risk pregnancies  *N*=101,963 (57.7%) | 11,252 (11.0%)  (95% CI 10.7-11.3%) | 840 (8.2)  (95% CI 7.5-9.0) | 2,005 (19.7)  (95% CI 18.5-20.9) | 2,845 (27.9)  (95% CI 26.6-29.3) |
| Percentage of outcome from low-risk pregnancies | 36.5% | 44.2% | 31.8% | 36.0% |

^a^: Unplanned c-sections are those that were not planned before the mother was in labor; ^b^: Stillbirths are pregnancies where the fetus dies after the mother’s 28^th^ week of pregnancy. Due to data collection structure of NFHS, multiple pregnancy, breech presentation, and no antenatal care by sixth month of pregnancy variables are not available for stillbirth calculation; ^c^: Neonatal deaths are deaths among live births during the first 28 completed days of life; ^d^: Composite is the summation of stillbirths and neonatal deaths. Data presented are the weighted estimates from the sample and rates are estimated per 1,000 live births along with 95% confidence intervals that account for the complex survey design.

**Table S3: Adverse birth outcomes by state**

| **State/union territory** | **Population**  **millions**  **(2020)** | ***Total*** | | | | |  | ***Among PMSMA low-risk pregnancies*** | | | | |
| --- | --- | --- | --- | --- | --- | --- | --- | --- | --- | --- | --- | --- |
|  |  | **Pregnancies** | **Unplanned**  **C-sections (Percent)** | **Stillbirths (per 1,000 live births)** | **Neonatal**  **Deaths (per 1,000 live births)** | **Composite (per 1,000 live births)** | **Pregnancies** | | **Unplanned C-sections (Percent)** | **Stillbirths (per 1,000 live births)** | **Neonatal deaths (per 1,000 live births)** | **Composite (per 1,000 live births)** |
| India | 1,347 | 176,699 | 10% | 8.5 | 16.6 | 25.2 | 101,347 | | 8% | 8.4 | 13.5 | 21.9 |
| Uttar Pradesh | 228 | 33,055 | 7% | 11.5 | 25.9 | 37.5 | 20,344 | | 6% | 11.1 | 22.2 | 33.2 |
| Maharashtra | 123 | 14,931 | 11% | 6.1 | 10.8 | 16.9 | 9,416 | | 10% | 5.6 | 8.2 | 13.8 |
| Bihar | 121 | 20,337 | 6% | 11.4 | 23.2 | 34.6 | 12,324 | | 4% | 10.8 | 17.5 | 28.3 |
| West Bengal | 98 | 13,836 | 14% | 9.8 | 10.6 | 20.4 | 6,061 | | 13% | 9.1 | 9.6 | 18.5 |
| Madhya Pradesh | 83 | 10,161 | 6% | 7.0 | 18.4 | 25.4 | 6,458 | | 5% | 5.6 | 13.8 | 19.4 |
| Rajasthan | 78 | 11,171 | 6% | 5.4 | 13.9 | 19.2 | 7,706 | | 5% | 4.5 | 10.8 | 15.3 |
| Tamil Nadu | 76 | 8,759 | 19% | 3.8 | 7.3 | 11.1 | 4,432 | | 16% | 3.6 | 3.6 | 7.0 |
| Gujarat | 69 | 7,622 | 10% | 6.3 | 14.8 | 21.1 | 4,658 | | 8% | 7.1 | 13.5 | 20.6 |
| Karnataka | 66 | 7,838 | 15% | 5.6 | 9.6 | 15.2 | 3,662 | | 14% | 5.7 | 7.6 | 13.7 |
| Andhra Pradesh | 53 | 5,456 | 15% | 8.2 | 14.1 | 22.4 | 2,518 | | 14% | 11.9 | 4.8 | 16.7 |
| Odisha | 44 | 6,164 | 11% | 11.0 | 17.4 | 28.4 | 3,748 | | 11% | 9.3 | 13.3 | 22.7 |
| Jharkhand | 38 | 5,239 | 7% | 10.9 | 21.6 | 32.3 | 3,200 | | 6% | 9.7 | 16.3 | 26.3 |
| Telangana | 37 | 3,856 | 23% | 7.0 | 13.0 | 20.0 | 1,733 | | 23% | 8.7 | 13.3 | 21.9 |
| Kerala | 35 | 3,950 | 11% | 3.0 | 2.5 | 5.8 | 1,169 | | 14% | 6.0 | 3.4 | 10.3 |
| Assam | 35 | 5,119 | 7% | 7.0 | 17.6 | 24.6 | 2,755 | | 7% | 7.3 | 12.3 | 19.6 |
| Punjab | 30 | 3,307 | 16% | 8.2 | 12.7 | 20.9 | 1,834 | | 15% | 9.3 | 7.6 | 16.9 |
| Chhattisgarh | 29 | 3,954 | 8% | 9.1 | 18.7 | 27.8 | 2,515 | | 8% | 10.3 | 11.5 | 21.9 |
| Haryana | 29 | 3,337 | 8% | 9.0 | 12.0 | 21.0 | 2,059 | | 7% | 8.3 | 10.7 | 18.9 |
| Delhi | 20 | 2,390 | 12% | 10.5 | 11.7 | 22.2 | 1,361 | | 10% | 9.6 | 8.8 | 17.6 |
| Jammu & Kashmir | 13 | 1,395 | 16% | 7.2 | 5.7 | 12.9 | 650 | | 14% | 10.8 | 4.6 | 15.4 |
| Uttarakhand | 11 | 1,445 | 9% | 10.4 | 21.5 | 31.8 | 922 | | 6% | 10.8 | 18.4 | 30.4 |
| All other states | 29 | 3,379 | 8% | 6.8 | 10.1 | 17.5 | 1,823 | | 8% | 6.6 | 8.2 | 15.4 |

Note: Data are census projections of population, weighted number of pregnancies, percent of unplanned C-sections, stillbirth rate, neonatal mortality rate and composite rate of adverse outcomes (stillbirths and neonatal deaths), both total and among ‘low-risk’ PMSMA pregnancies. Data are from NFHS-5 and account for the complex survey design. The census projections of population are from the 2011 census.
